# Effects of Exercise on Dopamine Expression and Motor Recovery in Hemorrhagic and Ischemic Stroke Rat Models With Comparable Brain Lesions

**DOI:** 10.7759/cureus.86395

**Published:** 2025-06-19

**Authors:** Keigo Tamakoshi, Kota Meguro, Yuri Takahashi, Ryu Oshimi, Natsuka Iwasaki

**Affiliations:** 1 Department of Physical Therapy, Niigata University of Health and Welfare, Niigata, JPN; 2 Department of Rehabilitation, Kaetsu Hospital, Niigata, JPN; 3 Department of Rehabilitation, Aizawa Hospital, Nagano, JPN; 4 Department of Rehabilitation, Saigata Medical Center, Niigata, JPN; 5 Department of Rehabilitation, Azuma Neurosurgical Hospital, Fukushima, JPN

**Keywords:** dopamine expression, intracerebral hemorrhage, ischemic stroke, motor function recovery, treadmill exercise

## Abstract

This study investigated the differential effects of identical treadmill exercise regimens on motor function recovery, lesion volume, and dopamine expression in rat models of ischemic stroke (ISC) and intracerebral hemorrhage (ICH) with comparable brain injury size and location. Thirty male Wistar rats were divided into five groups: ISC, ICH, sham (SHAM), ISC with exercise (ISC + Ex), and ICH with exercise (ICH + Ex). ISC and ICH lesions were induced in the left striatum using endothelin-1 and bacterial collagenase, respectively. Rats in the exercise groups underwent treadmill running (11 m/min, 30 min/day) from postoperative day 4 to 27. Motor function was assessed using the rotarod test, lesion volume was measured via Nissl staining, and dopamine expression was analyzed using tyrosine hydroxylase (TH) immunohistochemistry. On postoperative day 28, exercise significantly reduced lesion volume in ISC + Ex compared to ISC but had no effect on lesion volume in ICH + Ex. Motor function improved in ISC + Ex but not in ICH + Ex. However, TH expression was significantly higher in the ICH + Ex group than in other groups, suggesting that dopamine contributes to recovery mechanisms after ICH. Treadmill exercise had distinct effects on ISC and ICH recovery. In ISC, exercise improved motor function and reduced lesion volume. In ICH, it enhanced dopamine expression without improving motor function. These findings suggest that rehabilitation strategies should be tailored to stroke type to optimize recovery outcomes.

## Introduction

Stroke is divided into intracerebral hemorrhage (ICH) and ischemic stroke (ISC), with the latter being more common worldwide. Notably, ICH patients have a high mortality rate in the early stages of the disease [1,2]. One clinical study found that functional recovery was superior in patients with ICH versus ISC when the degree of brain damage was comparable [3,4]. Despite ICH having a higher mortality rate in the initial phase, its eventual functional recovery has been reported to be comparable to that of ISC, particularly in terms of functional status change at discharge from intensive rehabilitation hospitals, as shown in some studies [5]. Another study compared and verified the functional recovery of ICH and ISC in animal experiments, reporting that function recovered faster in the former [6]. We previously reported that these differences in functional recovery between the two models are likely attributed to differences in secondary degeneration and astrocytic responses [7,8]. These findings, along with previous reports, suggest that beyond the primary lesion, the underlying mechanisms of brain reorganization and repair, such as secondary degeneration and astrocytic responses, exhibit distinct differences between ISC and ICH.

Exercise therapy is known to improve outcomes after stroke by promoting neuroplasticity and limiting secondary brain damage. In particular, early exercise has been shown to reduce infarct volume in models of ISC, possibly through neuroprotective mechanisms [9,10]. However, similar volume-reducing effects of exercise have not been consistently observed in ICH models, despite evidence of functional improvement [11,12]. This discrepancy raises the question of whether different molecular or circuit-level mechanisms underlie the benefits of rehabilitation in ICH.

Dopamine, a key neuromodulator, is critically involved in motor control, motivation, and synaptic plasticity. Experimental evidence has shown that dopaminergic signaling contributes to neurorehabilitation by facilitating synaptic remodeling and functional reorganization in the injured brain [13,14]. Importantly, the striatum is often affected in both ISC and ICH, making it a relevant region for comparing dopaminergic responses to rehabilitation interventions.

Based on these findings, we hypothesized that the mechanisms of exercise-induced recovery differ between ISC and ICH. In ISC, exercise may reduce lesion volume and improve functional outcome through neuroprotective effects. In contrast, improvements in ICH may be driven by dopaminergic modulation of neural function rather than structural preservation.

To test this hypothesis, we applied the same exercise regimen to rat models of ISC and ICH with comparable striatal damage and assessed lesion volume, motor function, and dopaminergic activity in both groups.

## Materials and methods

Animals

Thirty male Wistar rats weighing between 200 and 300 grams were used in this experiment. The animals were randomly divided into five groups: ischemic surgery group (ISC, n = 6), hemorrhagic surgery group (ICH, n = 6), sham surgery group (SHAM, n = 6), post-ischemic exercise group (ISC + Ex, n = 6), and post-hemorrhagic exercise group (ICH + Ex, n = 6). A sham group was included to control for the potential effects of anesthesia, surgical stress, and handling, which could influence the outcomes independently of the experimental intervention. All outcome assessments were performed by investigators blinded to group assignments. The rats were housed in groups of three to four per clear Plexiglas cage (Ancare, USA), maintained at a constant room temperature of 23 ± 1°C, and subjected to a 12-hour light/dark cycle. Food and water were provided ad libitum. All procedures related to animal handling and surgery conformed to the institutional animal care guidelines of Niigata University of Health and Welfare (approval number 28005).

Surgery

Rats were anesthetized with an intraperitoneal injection of sodium pentobarbital (45 mg/kg) and then restrained in a stereotaxic apparatus. ISC or ICH brain lesions were induced in the left striatum according to established protocols [6-8]. For both procedures, a needle was inserted 3.6 mm lateral to the bregma and advanced 6.0 mm below the cranial surface. In the ICH group, 4 μL of sterile saline containing 50 units of bacterial collagenase (type IV, Sigma-Aldrich, St. Louis, MO, USA) was injected. For the ISC group, an equal volume of sterile saline containing 100 pmol endothelin-1 (human and porcine, Calbiochem, MilliporeSigma, Burlington, MA, USA) was administered. Rats in the SHAM group received a 4.0 μL injection of sterile saline without any drug.

Exercise

A treadmill device was used for the exercise intervention. For three days, starting four days before surgery, all rats were trained to run at progressively increasing speeds. From postoperative days 4 to 27, the rats ran at 11 m/min for 30 min once daily [11,12,15].

Behavioral testing

Motor function was assessed using the rotarod test [12,15]. Prior to surgery, all animals underwent pre-training on a rotarod apparatus set at a constant speed of 4 rpm, continuing until they were able to remain on the rotating rod for a minimum of 60 seconds. Subsequently, each animal performed five trials on an accelerating rotarod, in which the rotation speed increased linearly from 4 to 40 rpm over a period of five minutes. Behavioral assessments were conducted on postoperative days 1 and 28. All rotarod tests were conducted by a trained experimenter who was blinded to the experimental group assignments.

Lesion volume analysis

At 29 days post-surgery, rats were deeply anesthetized with sodium pentobarbital and subjected to transcardial perfusion with 0.9% saline followed by 4% paraformaldehyde in phosphate buffer (PB) (pH 7.4). Brains were subsequently extracted and post-fixed in the same paraformaldehyde solution. Coronal brain sections, 40 µm in thickness, were prepared using a freezing microtome, encompassing the bregma region from +1.9 mm to -0.1 mm. Six sections per animal were stained with Nissl solution. Lesion volumes within the striatum were quantified using ImageJ software (version 1.42, Wayne Rasband, National Institutes of Health (NIH), Bethesda, USA) and normalized to the volume of the contralateral hemisphere. For each of the six sections per animal, the lesion area within the striatum was manually outlined using the ImageJ software. The average area across these six sections was then calculated and used to represent the lesion extent. The lesion volume was then normalized to the volume of the contralateral hemisphere to account for potential brain swelling. The consistency of lesion location and extent across ISC and ICH groups was confirmed statistically (as reported in the Results section).

Immunohistochemistry

For immunohistochemical analysis, sections were processed with a rabbit polyclonal antibody against tyrosine hydroxylase (TH; 1:500, Merck, Darmstadt, Germany). Sections were initially blocked with a solution containing 5% normal goat serum in PB with 0.25% Triton X-100 for one hour. Subsequently, sections were incubated overnight at 4°C with the primary antibody, diluted in PB containing 0.25% Triton X-100 and 2.5% normal goat serum. Following rinsing, sections were incubated with a biotinylated goat anti-rabbit secondary antibody (Vector Laboratories, Burlingame, CA, USA), diluted 1:500 in PB with 0.3% Triton X-100, for two hours at room temperature. Antigen-antibody complexes were visualized using the avidin-biotin-peroxidase method (ABC Elite kit, Vector Laboratories) and reacted with a chromogen solution containing 0.025% diaminobenzidine tetrahydrochloride and 0.03% hydrogen peroxide in PB. For quantification of TH expression, optical density was measured using ImageJ software. Three non-overlapping, standardized regions of interest (100x100 µm squares) were randomly selected within the striatum of the lesioned hemisphere for each section, and their average optical density was calculated. Background optical density was measured from an unstained area within the corpus callosum of the same section and subtracted from the striatal measurements. All immunohistochemical analyses and optical density measurements were performed by an investigator blinded to the experimental group assignments.

Statistical analysis

Data are presented as mean ± standard error of the mean (SEM). Statistical analyses were performed using Microsoft Excel (Microsoft® Corp., Redmond, WA, USA) with the Bell Curve statistical plugin (Social Survey Research Information Co., Ltd., Tokyo, Japan). One-way analysis of variance (ANOVA) was employed to determine significant differences among experimental groups, followed by Tukey’s post hoc test for multiple comparisons when applicable.

## Results

Motor function recovery

**Figure 1:**
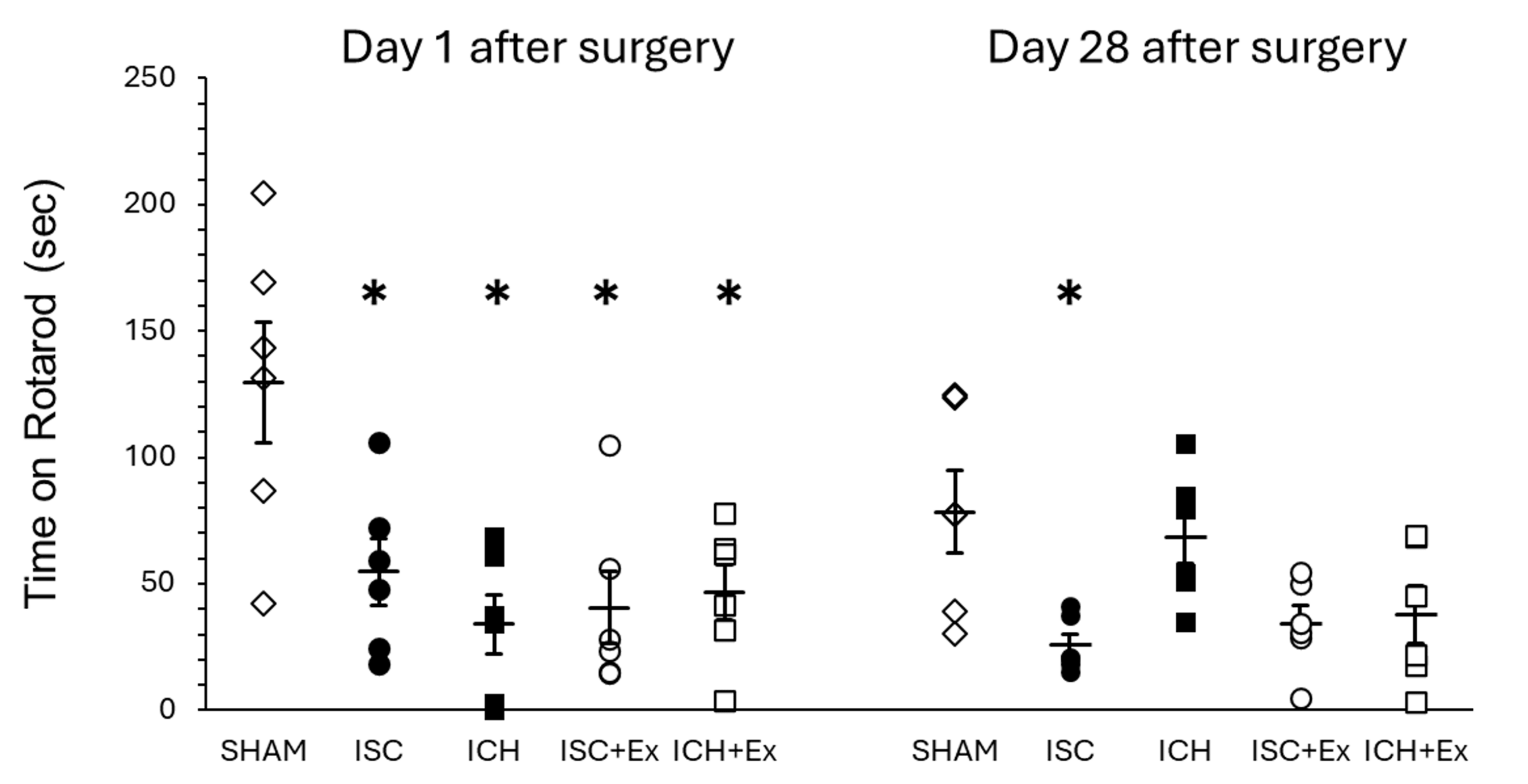
Rotarod test Rotarod performance (seconds) at one and 28 days post-surgery in sham-operated (SHAM), ischemic stroke (ISC), intracerebral hemorrhage (ICH), and exercise groups (ISC + Ex, ICH + Ex). At day 28, ISC showed significantly lower performance than SHAM, while ICH, ICH + Ex, and ISC + Ex were comparable to SHAM. * indicates P < 0.05 vs. SHAM.

Figure 1 illustrates the rotarod performance, quantified as the time spent on the apparatus (in seconds), for each experimental group at one and 28 days post-surgery. At the initial assessment (day 1), both the ISC and ICH groups exhibited a significant reduction in rotarod performance compared to the SHAM group, indicating acute motor deficits following stroke induction (*P < 0.05). By day 28, the ISC group continued to display significantly impaired motor function relative to the SHAM group (*P < 0.05). Notably, the exercise groups (ISC + Ex and ICH + Ex) showed a trend towards improved rotarod performance at day 28. These findings suggest a potential beneficial effect of exercise on motor recovery, particularly within the ISC model.

Lesion volume

**Figure 2:**
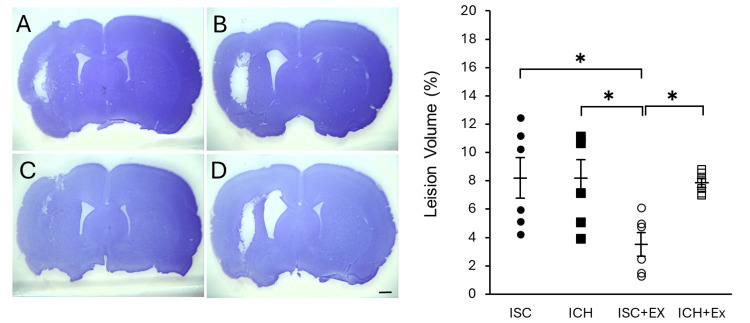
Lesion volume This figure illustrates the effect of exercise on lesion volume in ISC and ICH models, as assessed by Nissl staining. Figures A-D show Nissl-stained brain tissue sections from each experimental group: (A) ISC, (B) ICH, (C) ISC + Ex, and (D) ICH + Ex. The accompanying graph quantitatively compares lesion volume (%) between these groups. Notably, the ISC + Ex group showed a significantly reduced lesion volume compared to the ISC group, with a statistically significant difference. * indicates P < 0.05. ISC: ischemic stroke; ICH: intracerebral hemorrhage; Ex: exercise group

Nissl staining was employed to quantify lesion volume (%) as a measure of brain damage in ISC and ICH model animals. To ensure comparability of lesion severity, both ISC and ICH groups were established with controlled lesion size and location, resulting in comparable lesion volumes between the two groups. Notably, the ISC + Ex group demonstrated a significantly reduced lesion volume compared to the ISC group (*P < 0.05), suggesting a neuroprotective effect of exercise following ISC. No significant difference in lesion volume was observed between the ICH and ICH + Ex groups. These findings suggest that exercise may attenuate brain damage following stroke, with a potential differential effect based on stroke type (Figure 2).

TH expression

**Figure 3:**
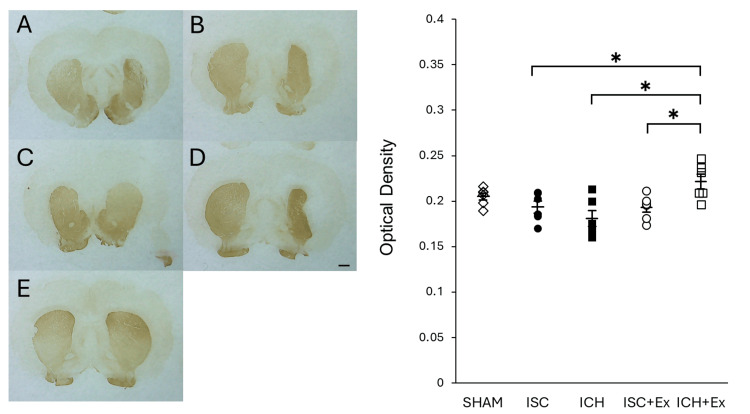
Tyrosine hydroxylase (TH) immunostaining in the striatum Immunohistochemical staining for TH in the striatum, along with corresponding quantification of TH optical density, is shown for each experimental group. Representative coronal brain sections (A-E) illustrate TH staining patterns: (A) ISC, (B) ICH, (C) ISC + Ex, (D) ICH + Ex, and (E) SHAM. The graph quantifies the optical density of TH in the striatum. Notably, the ICH + Ex group exhibited a significantly higher TH optical density compared to the ICH group. * indicates P < 0.05. ISC: ischemic stroke; ICH: intracerebral hemorrhage; Ex: exercise group; SHAM: sham surgery group

Dopamine expression activation in each experimental group was assessed via immunohistochemical staining and optical density measurement of TH in the striatum. The ICH + Ex group exhibited a significantly higher TH optical density in the striatum compared to both the ICH and ISC + Ex groups (Figure 3, *P < 0.05). This finding suggests that exercise therapy may facilitate TH expression, a proxy for dopaminergic tone, following activation by ICH.

## Discussion

This study examined the effects of identical exercise conditions in rat models of cerebral hemorrhage and infarction with brain injuries of similar location and size. In terms of motor function, the process of spontaneous recovery was faster in cerebral hemorrhage compared to infarction, but exercise had no statistically significant effect on motor function recovery in this group. However, exercise tended to improve motor dysfunction in cerebral infarction, which could be attributed to the decrease in lesion volume after exercise. In cerebral hemorrhage, motor function did not improve after exercise, but dopamine expression in the striatum was increased.

Exercise has a beneficial, multifaceted impact on recovery in the ISC rat model, significantly decreasing infarct volume, cell death, and neurological deficits [10]. Treadmill exercise promotes dendritic and synaptic plasticity in the penumbra region, which is crucial for neurological recovery. This effect is mediated through the upregulation of the caveolin-1/vascular endothelial growth factor (VEGF) signaling pathways [16]. Additionally, treadmill exercise also improves reperfusion injury in cerebral ischemia by enhancing interleukin 4 (IL-4) expression and promoting M2 microglia activation, potentially through the JAK1-STAT6 pathway [17]. These studies suggest that treadmill exercise after stroke in the cerebral ischemia rat model can improve neurobehavioral outcomes, reduce infarct volumes, enhance motor functions, promote neurogenesis and synaptic plasticity, and modulate inflammatory responses. A similar mechanism likely occurred in this study, since exercise was found to reduce the lesion volume in the infarct group. Conversely, the beneficial effects of exercise have also been reported in cerebral hemorrhage. Although treadmill exercise does not reduce the lesion volume, it inhibits dendritic regression and increases TrkB protein expression [11,18]. Specifically, the inhibition of dendritic regression could be attributed to the suppression of IL-1b protein expression, which is usually increased after cerebral hemorrhage [12]. Nevertheless, neither simple exercises nor complex exercises have been shown to reduce the brain damage caused by hemorrhage [19].

The effects of exercise after cerebral hemorrhage on dopamine have not been previously reported, but several reports have discussed this in cerebral ischemia. Exercise has demonstrated beneficial effects on cognitive function and anxiety-like behavior in rat models of cerebral ischemia. Specifically, pre-ischemic voluntary wheel running increased basal striatal dopamine levels and attenuated the ischemia-induced dopamine surge, which led to improved cognitive function and reduced anxiety [20,21]. Another study found that treadmill exercise after neonatal hypoxic-ischemic brain injury enhanced dopamine expression in the substantia nigra and striatum, thereby ameliorating spatial learning deficits. However, our results suggest the involvement of dopamine in the improvement of motor dysfunction caused by exercise after cerebral hemorrhage. Dopamine is a major neurotransmitter in the central nervous system, which is crucial in motor learning and neuroplasticity, and its involvement in the recovery of motor function after stroke has previously been suggested [22-24]. In animal models, the stimulation of dopamine receptors promotes synaptic plasticity, which helps in learning new motor skills [25] and supports the recovery of motor function through the reorganization of neural circuits [26]. These differences can be attributed to the various genetic backgrounds and neurological conditions of the individual patients. For example, polymorphisms in the COMT gene, which is involved in the metabolism of dopamine, have been suggested to influence the recovery of motor function, which could in turn influence treatment outcomes [27]. In summary, the current evidence suggests that dopamine is one of the key factors supporting the recovery of motor function, but its effects vary considerably based on the genetic background and pathology of the patient. Future research is needed to optimize the use of dopamine agonists and rehabilitation strategies.

The results of these previous studies cannot be directly compared due to the differences in the location and size of the ischemic or hemorrhagic brain injury, as well as the differences in exercise conditions. However, in the present study, an identical exercise regimen was administered across all rats with the same site and size of brain injury (hemorrhagic or ischemic), thereby allowing for a comparison of the exercise effects between the two groups. The results of this study may provide useful data for reconsidering the treatment strategy in stroke rehabilitation, since the effects of exercise differed between cerebral ischemia and hemorrhage.

The study has several limitations that should be acknowledged. First, although the lesion size and location were standardized in this study, our results may not be applicable to other injuries of different sizes or locations. Further research is needed to investigate the effects of exercise across different stroke models. Second, the assessment of motor function relied solely on the rotarod test, which assessed whole-body balance but did not directly measure motor paralysis or specific functional impairments. Thus, expanding the range of tests may provide a more nuanced and comprehensive understanding of recovery. Third, the results are based on a single type of exercise with fixed conditions, which does not fully reflect the diversity of rehabilitative exercises used in clinical settings. Future studies should explore different exercise types, intensities, and durations to optimize rehabilitation strategies. Fourth, although dopamine expression was analyzed, the precise mechanisms behind its role in recovery, particularly its causal link to functional recovery, remain unclear. This study observed increased dopamine expression but did not include pharmacological or genetic interventions to directly test the causal role of dopaminergic signaling in functional recovery. Therefore, further molecular studies incorporating dopamine receptor blockade or pathway-specific manipulations will be necessary to validate the mechanistic involvement of dopamine in exercise-induced recovery. Fifth, the study did not address potential differences in age or sex between the animal models, which can also influence recovery and response to rehabilitation. Finally, the findings in this animal model may not be fully applicable in human patients due to species-specific differences in stroke pathology and recovery processes. These limitations suggest the need for more comprehensive and translational research to advance the understanding of exercise-based stroke rehabilitation.

## Conclusions

This study demonstrates that an identical treadmill exercise regimen yields distinct outcomes in motor recovery and brain changes in rat models of ISC and ICH, even when initial brain lesion size and location are comparable. Specifically, in ISC models, exercise significantly improved motor function and reduced lesion volume, suggesting a neuroprotective effect that contributes to functional recovery. In contrast, in ICH models, exercise did not improve motor function but markedly enhanced striatal dopamine expression, as indicated by TH levels. This novel finding indicates that while direct functional improvement was not observed in ICH, exercise may still modulate neurochemical pathways, specifically dopaminergic signaling, which could play a role in underlying recovery mechanisms after hemorrhagic stroke.

These differential effects underscore the critical need for tailoring rehabilitation strategies to the specific type of stroke. Future research should investigate the precise mechanisms by which exercise influences dopamine expression in ICH and explore whether targeted interventions can leverage this neurochemical modulation to enhance motor recovery in hemorrhagic stroke patients. Furthermore, expanding behavioral assessments beyond the rotarod test and investigating different exercise parameters could provide a more comprehensive understanding of exercise's impact on stroke recovery.
